# Knowledge and Confidence of Obstetrics and Gynecology Residents in the Evaluation and Management of Heavy Menstrual Bleeding Due to Inherited Bleeding Disorders

**DOI:** 10.1089/whr.2024.0086

**Published:** 2024-09-24

**Authors:** Patricia S. Huguelet, Irmel A. Ayala, Laurel Beaty, Christina Bemrich-Stolz, Claudia Borzutzky, Tazim Dowlut-McElroy, Sweta Gupta, Kendra Hutchens, Corinna L. Schultz, Lakshmi Srivaths, Maria C. Velez, Neeraja Swaminathan

**Affiliations:** ^1^ Department of Obstetrics and Gynecology, Section of Pediatric and Adolescent Gynecology, University of Colorado Anschutz Medical Campus and Children’s Hospital Colorado, Aurora, Colorado, USA.; ^2^ Division of Hematology, Cancer and Blood Disorder Institute, Johns Hopkins All Children’s Hospital, St. Petersburg, Florida, USA.; ^3^ Department of Biostatistics and Informatics, University of Colorado Anschutz Medical Campus Aurora, Colorado, USA.; ^4^ Division of Pediatric Hematology-Oncology, Department of Pediatrics, University of Alabama at Birmingham, Birmingham, Alabama, USA.; ^5^ Division of Adolescent and Young Adult Medicine, Department of Pediatrics, Keck School of Medicine of University of Southern California and Children’s Hospital Los Angeles, Los Angeles, California, USA.; ^6^ Division of Pediatric and Adolescent Gynecology, Department of Surgery, Children’s Mercy Hospital, Kansas City, Missouri, USA.; ^7^ Indiana Hemophilia and Thrombosis Center, Indianapolis, Indiana, USA.; ^8^ Lisa Dean Moseley Foundation Institute for Cancer and Blood Disorders, Nemours Children’s Health, Jacksonville, Florida, USA.; ^9^ Department of Pediatric Hematology-Oncology, University of Texas Health Sciences Center-Houston, Houston, Texas, USA.; ^10^ Division of Hematology-Oncology, Department of Pediatrics, Louisiana State University Health Sciences Center and Children’s Hospital New Orleans, New Orleans, Louisiana, USA.; ^11^ Division of Hematology-Oncology, Department of Pediatrics, University of Michigan Medical School, Ann Arbor, Michigan, USA.

**Keywords:** heavy menstrual bleeding, inherited bleeding disorders, medical education

## Abstract

**Background:**

Heavy menstrual bleeding (HMB) is common, and 20–30% of patients presenting with HMB are diagnosed with an inherited bleeding disorder (IBD). Despite the frequent association of HMB with bleeding disorders, specific learning objectives on this topic are lacking for Obstetrics and Gynecology (OBGYN) residents.

**Objective:**

We sought to determine the exposure of OBGYN residents to didactics, clinical training, and confidence in evaluation and management of patients with HMB due to IBDs.

**Methods:**

Prospective survey of OBGYN residents through email solicitation. Residents were invited to complete an anonymous 26-item survey, querying residents’ confidence in evaluation and management of HMB in patients with and without IBDs.

**Results:**

In total, 239 OBGYN residency programs were invited to participate and 20 programs responded. Among 388 residents, 84 completed the survey (21.6%). The majority reported didactics on HMB evaluation (*n* = 71, 85.5%) and treatment (*n* = 77, 92.8%); however, for HMB due to IBDs, only 35 residents (42.4%) reported didactics on evaluation and 28 (33.7%) reported didactics on treatment. Confidence in evaluation and management of HMB was high but decreased significantly with an IBD. Residents who received didactics on IBDs reported more confidence in their evaluation than residents who did not receive didactics (mean Likert scale score of 3.67 vs. 3.23, *p* = 0.002). Increasing postgraduate year level was associated with more confidence in treatment (*p* < 0.001) and did not differ based on type of training program (*p* = 0.825).

**Conclusion:**

OBGYN residents have decreased confidence in evaluation and management of HMB due to IBDs. Resident confidence increases with didactics and training. Residents would benefit from curricula designed to address this deficit in training.

## Introduction

Abnormal uterine bleeding (AUB), including heavy menstrual bleeding (HMB), is common, occurring in approximately 20%–40% of reproductive-aged females, and accounts for nearly one-third of outpatient visits to gynecologists.^1–4^ The etiology of AUB is multifactorial and commonly described using the PALM-COEIN index: Polyp, Adenomyosis, Leiomyoma, Malignancy and hyperplasia, Coagulopathy, Ovulatory dysfunction, Endometrial, Iatrogenic, and Not otherwise classified.^3^ In adolescents, while the majority of abnormal bleeding is due to anovulatory cycles, it has recently been recognized that up to 62% of adolescents experiencing HMB may have an underlying inherited bleeding disorder (IBD).^5^ When evaluating females of all ages presenting with HMB, IBDs have been identified in up to 30% of patients.^6–8^

Females with IBDs experience a wide spectrum of obstetric and gynecological bleeding symptoms resulting in substantial morbidity, mortality, and poor quality of life.^6,9^ Studies have shown that adolescents and young adults with IBDs can experience bleeding complications resulting in severe anemia, hypovolemic shock, inpatient hospitalization, and coagulopathy.^10–12^ Despite the significant negative impact of IBDs, several studies have underscored that females with IBDs experience barriers to optimal care.^13–15^ Improving provider education is key to ensuring appropriate care, timely referral, and optimal management for these patients.

The Council for Resident Education in Obstetrics and Gynecology establishes learning objectives for Obstetrics and Gynecology (OBGYN) residents to master during training.^16^ Despite the high prevalence of IBDs in adolescents and adult women, specific learning objectives on this topic are lacking. There is also a paucity of data evaluating whether OBGYN training programs adequately prepare residents to identify and manage patients with IBDs. One previous study evaluated the educational approach to IBDs in OBGYN residents, concluding that a better approach is needed.^17^ However, this survey only queried chief residents and did not assess confidence in training or whether a correlation existed between educational exposure and confidence in clinical practice. In addition, this study did not offer any educational tools to address the deficiencies described in their study.^17^

We therefore sought to assess OBGYN resident exposure to educational didactic sessions, clinical cases, and overall confidence in the evaluation and management of patients with HMB both with and without IBDs, across the 4-year training program. We further propose an educational curriculum with a set of educational tools and resources to address the deficiencies we identified.

## Materials and Methods

After obtaining Institutional Review Board exemption through the University of Michigan, we prospectively invited 239 U.S. OBGYN residency programs to participate in a research study assessing resident training on the topic of inherited blood disorders. Research participation was solicited through an email inquiry to program directors. Program directors were informed that the study would be anonymous and optional for all resident participants. If interested, program directors then distributed the email inquiry directly to their respective residents. Program directors were sent three monthly reminder emails to solicit participation in the study.

The study was conducted from February 2022 to May 2022. Within the email inquiry, resident participants were provided with a secure link to an online Research Electronic Data Capture (REDCap) questionnaire. REDCap is an electronic data capture tool hosted by the University of Colorado. All data were stored in this secure database and subsequently analyzed using R version 4.2.2.

The REDCap questionnaire consisted of 26 items that inquired about OBGYN resident knowledge and clinical and didactic exposure to the topic of HMB in patients with and without IBDs. A five-point Likert scale was used to query resident confidence in the evaluation and management of HMB and iron deficiency anemia in patients with and without suspected IBDs. Additional items surveyed included exposure to clinical cases, lectures, or other structured educational sessions on these topics. Basic demographic information, including year of postgraduate training, gender, and type of training program (academic vs. community), was also collected.

Descriptive statistics were used to analyze continuous variables using means and ranges for continuous variables and counts and proportions for categorical variables. To estimate average confidence, we assigned a numeric point value to the Likert scale (*i.e.,* very unconfident = 1 and extremely confident = 5). For tests of association with residency year, we used linear analysis of variance, and for tests comparing training and program with confidence, we used independent *t*-tests. To compare confidence in the evaluation and management of HMB with confidence specifically in the evaluation and management of HMB due to IBDs, we used a paired *t-*test. To compare the proportion of binary responses to specific questions, we used McNemar’s chi-square test with a continuity correction.

## Results

We received email responses from 20 programs; among 388 residents at those programs, 84 completed the survey (21.6% response rate). Respondents matriculated at both academic (54.2%) and community (45.8%) programs and represented most geographic locations in the United States, including the Southeast, Northeast, Midwest, and Western regions. Residents from all training levels (PGY 1–4) participated, and the majority were female (95.2%) (Table 1).

**Table 1.** Demographic Characteristics

Demographic	Total *N* = 83 (%)
PGY Level	
PGY-1	21 (25.3)
PGY-2	27 (32.5)
PGY-3	26 (31.3)
PGY-4	9 (10.8)
Program	
Academic	45 (54.2)
Community	38 (45.8)
Sex	
Female	79 (95.2)
Male	4 (4.8)

PGY, postgraduate year.

**FIG. 1.**
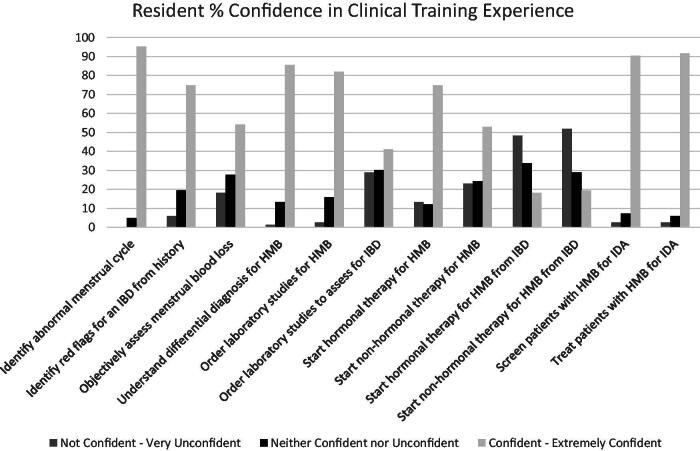
Resident confidence in clinical training experience.

Most participants reported receiving didactics on the evaluation (*n* = 71, 85.5%) and treatment (*n* = 77, 92.8%) of HMB. However, when we inquired specifically about didactics on the evaluation and treatment of HMB due to an IBD, only 35 residents (42.4%) reported receiving didactics on evaluation and 28 (33.7%) on treatment. Additionally, OBGYN resident confidence in the evaluation and management of HMB was high at baseline but decreased significantly in the setting of an IBD (Fig. 1).

When we analyzed resident confidence levels in the evaluation of patients with HMB in general compared with the evaluation of a patient with HMB specifically due to a bleeding disorder, resident confidence was lower in the setting of an IBD (3.97 vs. 3.25, *p* < 0.001). When we further analyzed specific topic areas in the evaluation and management of HMB, residents were less confident ordering laboratory tests for hemostatic evaluation, initiating hormonal treatment in patients with HMB, and initiating nonhormonal treatment in the setting of an IBD (*p* < 0.001, Table 2).

**Table 2.** Comparison of Confidence Levels in Residents Evaluating Patients with Heavy Menstrual Bleeding in Specific Topic Areas

Specific topic areas (*N* = 83)	Self-reported “confident” or “extremely confident”*n* (%)	Self-reported “neither/not/very unconfident *n* (%)	*p* Value
Residents who feel confident ordering laboratory studies and imaging for causes of heavy menstrual bleeding	68 (81.9)	15 (18.1)	*p* < 0.001
Residents who feel confident ordering laboratory studies to evaluate for a **bleeding disorder** in patients with heavy menstrual bleeding	34 (41.0)	49 (59.0)	

Residents who feel confident initiating hormonal treatment in a patient with heavy menstrual bleeding	62 (74.7)	21 (25.3)	*p* < 0.001
Residents who feel confident initiating hormonal treatment in a patient with heavy menstrual bleeding specifically due to a **bleeding disorder**	15 (18.1)	68 (81.9)	

Residents who feel confident initiating nonhormonal treatment in a patient with heavy menstrual bleeding	44 (53.0)	39 (47.0)	*p* < 0.001
Residents who feel confident initiating hormonal treatment in a patient with heavy menstrual bleeding specifically due to a **bleeding disorder**	16 (19.3)	67 (80.7)	

Residents who received didactics in both the evaluation and treatment of patients with HMB due to IBDs reported more confidence in their evaluation of those patients, compared with those who did not receive didactics (*p* < 0.001; Table 3). Increasing level of residency training was associated with more confidence in the management of these patients (*p* < 0.001), and the confidence level did not differ based on type of training program (Table 4).

**Table 3.** Relationship Between Resident Training and Confidence

Teaching/didactics (*N* = 83)	Self-reported confidence (mean, SD)	*p* Value
Received didactics in the clinical assessment and evaluation of patients with HMB due to a bleeding disorder		
Yes (*n* = 35)	3.67 (0.53)	*p* = 0.002
No (*n* = 48)	3.23 (0.66)	
Received didactics on how to manage HMB due to a bleeding disorder		
Yes (*n* = 28)	3.75 (0.53)	*p* < 0.001
No (*n* = 55)	3.24 (0.64)	

HMB, heavy menstrual bleeding; IBD, inherited bleeding disorder.

**Table 4.** Relationship Between Year of Training, Training Program, and Resident Confidence

Residency training (*N* = 83)	Self-reported confidence (mean, SD)	*p* Value
Was there an association between year of residency training and resident confidence		
PGY-1 (*n* = 21)	3.33 (0.37)	*p* < 0.001
PGY-2 (*n* = 27)	3.74 (0.47)	
PGY-3 (*n* = 26)	4.01 (0.54)	
PYG-4 (*n* = 9)	4.33 (0.43)	
Was there an association between academic and community program and resident confidence		
Academic (*n* = 45)	3.77 (0.58)	*p* = 0.825
Community (*n* = 38)	3.80 (0.54)	

PGY, postgraduate year.

## Discussion

Adolescents and adult women with IBDs experience excessive obstetric and gynecological bleeding symptoms that adversely affect their quality of life.^5,9,18,19^ While HMB is the most common bleeding symptom occurring in approximately 75–80% of females with IBDs, these patients can also have ovulation bleeding, hemoperitoneum, miscarriages, and surgical bleeding.^20–21^ In the last decade, there has been increasing awareness that if left unrecognized, bleeding in females with IBDs can result in life-threatening complications and even death.^10,22^ Several studies, including one from the Centers for Disease Control and Prevention, have shown that females with IBDs are often not diagnosed until adulthood, although they have had bleeding symptoms since adolescence.^23–24^ A study comparing the bleeding phenotypes and interventions between adolescents and adult women highlighted that adults were more likely to be diagnosed with a bleeding disorder compared with adolescents, potentially due to the common occurrence of HMB from an immature hypothalamic–pituitary–ovarian axis, thereby preventing the physician from considering an underlying IBD.^25^ The American College of Obstetricians and Gynecologists’ Committee Opinion from 2019 and the Foundation for the Women and Girls with Blood Disorders both underscore the importance of multidisciplinary care involving collaboration between hematologists and gynecologists, to provide specialized care for these complex patients.^20,26^

Several studies have highlighted the barriers to optimal care faced by females with IBDs across the world.^15,27–29^ A recent study showed that poor awareness by healthcare providers regarding IBDs emerged as one of the key gaps, amid other barriers including poor patient health literacy and limited access to care.^12^ Although it has been previously demonstrated that OBGYNs need more education about IBDs,^30^ studies assessing the educational curricula of OBGYN trainees in the United States are limited.^17^ Because OBGYNs are frequently the first point of contact for patients presenting with HMB, educating OBGYN trainees in the evaluation and treatment of patients with IBDs is crucial. Our study aimed to address this knowledge gap and provide insights about the training curricula of OBGYN residents specific to patients with IBDs.

In our current study, OBGYN residents demonstrated high levels of self-reported knowledge and confidence in the general assessment of HMB, but this significantly decreased when queried about the evaluation and management of patients with IBDs. The majority of residents (82%) reported confidence in ordering laboratory testing in the setting of HMB, but this dropped to 41% when specifically ordering testing in the setting of a suspected bleeding disorder. This is consistent with a previously published survey of OBGYN residents in which study participants were presented with a clinical scenario strongly suggestive of an IBD and in which 98.5% of residents recommended a complete blood count as part of the evaluation, but only 23.1% recommended testing for von Willebrand factor levels and only 9.2% recommended a Factor VIII level.^17^ Of particular concern in our study was that <20% of OBGYN residents reported confidence in both hormonal and nonhormonal management of HMB in the setting of an IBD.

Our study demonstrates a positive relationship between focused education about HMB and IBDs and confidence in managing these patients. It similarly demonstrates that lack of focused didactics in IBDs correlates with a lack of confidence in managing these patients. Given that OBGYNs are likely to be the first point of contact for a substantial number of patients with HMB due to underlying IBD, improving resident education about IBDs is critical and will likely lead to improved clinical management of patients with IBDs. The Foundation for Women and Girls with Blood Disorders (FWGBD) advocates for the incorporation of specific learning objectives related to IBDs into the designated learning objectives of OBGYN residents.^31^ The Committee for Education and Advocacy within FWGBD recently organized a panel of experts to develop a focused curriculum for OBGYN residents to address IBD educational needs. The expert panel, consisting of 10 adult and pediatric hematologists and gynecologists with clinical and research expertise in IBDs, utilized the most recent Council on Resident Education in Obstetrics and Gynecology learning objectives,^16^ combined with the results of this study, to develop a targeted yet comprehensive educational syllabus for OBGYN residents. The topics review both adolescents and adults and include (1) etiology of HMB, (2) screening tools for HMB and suspected IBDs, (3) laboratory testing for patients with and without suspected IBDs, (4) hormonal and nonhormonal management of HMB and IBDs, (5) screening and treatment for iron deficiency anemia, and (6) impact of HMB on quality of life. The curriculum is included in Supplementary Appendix SA1 and can be found online at https://www.fwgbd.org, under their listed educational resources.^31^

Our results demonstrate a significant deficit in current OBGYN resident knowledge and confidence in the evaluation and management of patients presenting with HMB due to IBDs. We propose a novel curriculum to supplement current OBGYN training programs to address this deficit and improve the screening and treatment of adolescents and adults with HMB due to IBDs. Future research is needed to assess (1) the feasibility of adding the proposed educational curriculum to OBGYN training programs, (2) whether this positively impacts resident knowledge and confidence and ultimately, and (3) whether this translates to improved screening, diagnosis, and management of HMB in patients with IBDs. Furthermore, research is also needed in other subspecialties including pediatrics, family medicine, and hematology to assess whether similar deficits in training exist and to develop strategies to address them.

Limitations of our study include the small sample size with only 20 OBGYN programs responding to our survey, although there was a balanced response from both academic and community programs. There is also potential selection bias, with only residents with greater interest in the topic choosing to participate in our research study. Furthermore, these data are self-reported; findings only reflect resident perceptions of their confidence and knowledge, not actual knowledge and clinical proficiencies in the topics of interest. An additional limitation of this study is that the authors rely only on the reports of the residents surveyed for information about the didactic curricula offered at their programs, rather than surveying the program directors themselves. Finally, it should be noted that half of our survey participants were in the spring of their first 2 years of residency training. It is possible that additional knowledge and confidence in this topic area would be demonstrated if more respondents were in their third and fourth years of training. However, despite these limitations, the study reinforces previously published literature on the deficits in OBGYN resident education and is novel in our proposal of a curriculum to help address this unmet need.

## Conclusions

OBGYN residents have decreased exposure and confidence in the evaluation and management of HMB due to IBDs. Resident confidence increases with didactic education and training. Residents would benefit from specific curricula designed to address this deficit in training. Addressing this knowledge gap is a key step toward optimal diagnosis and management of HMB due to IBDs as OBGYN providers are frequently the first point of contact.

## Supplementary Material

Supplementary Appendix SA1

## Abbreviations Used

AUB: abnormal uterine bleeding
FWGBD: Foundation for Women and Girls with Blood Disorders
HMB: heavy menstrual bleeding
IBD: inherited blood disorder
OBGYN: Obstetrics and Gynecology
PALM-COEIN: polyp, adenomyosis, leiomyoma, malignancy and hyperplasia, coagulopathy, ovulatory dysfunction, endometrial, iatrogenic, not otherwise specified
PGY: postgraduate year
REDCap: Research Electronic Data Capture
