# Supplementary material for: Knowledge and Confidence of Obstetrics and Gynecology Residents in the Evaluation and Management of Heavy Menstrual Bleeding Due to Inherited Bleeding Disorders
Source: Womens Health Rep (New Rochelle). 2024 Sep 24;5(1):705–11. doi: 10.1089/whr.2024.0086 (PMC11491577; doi:10.1089/whr.2024.0086)
Supplement: Supplementary Appendix SA1 [file whr.2024.0086_supp_appendix_sa1.pdf]

Heavy Menstrual Bleeding and Inherited Blood Disorders

Obstetrics and Gynecology Residency Reading Curriculum

The objective of this reading curriculum is to provide the essential resources needed for the Obstetrics and Gynecology resident to acquire fundamental clinical knowledge on the topic of heavy menstrual bleeding (HMB) due to inherited blood disorders. The reading materials review the etiology of HMB due to blood disorders, recommended screening and diagnostic tools, and hormonal and non-hormonal management of this condition, including screening and treatment of iron deficiency anemia.

Each topic first provides the ***recommended reading*** articles, followed by ***supplemental reading*** materials for the trainee who desires more in-depth knowledge. Links to multi-media learning tools, including podcasts and webinars, are provided where available.

Table of Contents

Etiology of Heavy Menstrual Bleeding in Adolescents ---- 2

Etiology of Heavy Menstrual Bleeding in Adults ---- 2

Screening Tools for Heavy Menstrual Bleeding ---- 3

Diagnostic Evaluation of Heavy Menstrual Bleeding in Adolescents ---- 4

Diagnostic Evaluation of Heavy Menstrual Bleeding in Adults ---- 4

Laboratory Testing for Bleeding Disorders ---- 4

Screening for Iron Deficiency Anemia ---- 5

Oral and IV Iron Therapy for Iron Deficiency Anemia ---- 5

Hormonal Management of Heavy Menstrual Bleeding in Adolescents and Adults ---- 6

Non-Hormonal Management of Heavy Menstrual Bleeding in Adolescents and Adults ---- 6

Heavy Menstrual Bleeding and Quality of Life ---- 7

Attribution

Obstetrics and Gynecology Residency Reading Curriculum – Heavy Menstrual Bleeding and Inherited Blood Disorders was developed by the Education and Advocacy Subcommittee, a section of the Women/Girls with Bleeding Disorders Learning Action Network (WGBD LAN), supported by the Foundation for Women & Girls[+] with Blood Disorders.

The following experts and subcommittee members compiled the recommendations:
Tricia Huguelet, MD, Neeraja Swaminathan, MD, Claudia Borzutzky, MD, Sweta Gupta, MD, Irmel Ayala, MD, Nefertiti Durant, MD, Maria Velez, MD, and Christina Bemrich-Stolz, MD.

+ = includes people with the propensity to menstruate

Published: July 2023
Review and Update: due July 2025

Etiology of Heavy Menstrual Bleeding in Adolescents

Recommended Reading

- Hernandez A, Dietrich JE. Abnormal uterine bleeding in the adolescent. *Obstet Gynecol.* 2020; 135:615-21.
- Haamid F, Sass AE, Dietrich JE. Heavy menstrual bleeding in adolescents. *J Pediatr Adolesc Gynecol.* 2017: 30:335-40.
- Graham RA, Davis JA, Corrales-Medina FF. The adolescent with menorrhagia: Diagnostic approach to a suspected bleeding disorder. *Pediatr Rev.* 2018 Dec; 39(12):588-600.
- Díaz R, Dietrich JE, Mahoney D Jr, Yee DL, Srivaths LV. Hemostatic abnormalities in young females with heavy menstrual bleeding. *J Pediatr Adolesc Gynecol.* 2014 Dec; 27(6):324-9.
- Zia A, Lau M. Chapter 1: Evaluation of the Adolescent with Heavy Menstrual Bleeding. In: Srivaths LV (eds) *Hematology in the Adolescent Female.* 1st ed. Springer, Cham; 2020: 3-11.

Supplemental Reading

- James AH, Manco-Johnson MJ, Yawn BP, Dietrich JE, Nichols WL. Von Willebrand disease: key points from the 2008 National Heart, Lung, and Blood Institute guidelines. *Obstet Gynecol.* 2009 Sep; 114(3):674-678.
- Srivaths LV, Minard CG, O'Brien SH, Wheeler AP, Mullins E, Sharma M, et al. The spectrum and severity of bleeding in adolescents with low von Willebrand factor-associated heavy menstrual bleeding. *Blood Adv.* 2020 Jul 14; 4(13):3209-3216.
- Haley K. Platelet Disorders. *Pediatr Rev* 2020 May; 41(5):224-235.Venkateswaran L, Yee DL. Rare bleeding disorders in young women. *J Pediatr Adolesc Gynecol.* 2010 Dec;23(6 Suppl):S38-42.
- Soni H, Kurkowski J, Guffey D, Dietrich JE, Srivaths LV. Gynecologic bleeding complications in postmenarchal female adolescents receiving antithrombotic medications. *J Pediatr Adolesc Gynecol.* 2018 Jun; 31(3):242-246.
- Srivaths LV, Zhang QC, Byams VR, Dietrich JE, James AH, Kouides PA, Kulkarni R; Hemophilia Treatment Centers Network Investigators. Differences in bleeding phenotype and provider interventions in postmenarchal adolescents when compared to adult women with bleeding disorders and heavy menstrual bleeding. *Haemophilia.* 2018 Jan; 24(1):63-69.

Additional Learning Materials

7-2-1 Podcast: Normal Vs. Heavy Menstrual Bleeding

Etiology of Heavy Menstrual Bleeding in Adults

Recommended Reading

- James AH, Kouides PA, Abdul-Kadir R, Dietrich JE, Edlund M, Federici AB, et al. Evaluation and management of acute menorrhagia in women with and without underlying bleeding disorders: consensus from an international expert panel. *Eur J Obstet Gynecol Reprod Biol.* 2011; 158:124-34.
- ACOG Practice Bulletin 128: Diagnosis of Abnormal Uterine Bleeding in Reproductive-Aged Women. *Obstet Gynecol.* 2012; 120:197-206.

Supplemental Reading

- Kouides PA. von Willebrand disease and other disorders of hemostasis in the patient with menorrhagia. *Women's Health(Lond).* 2005 Sep; 1(2):231-44.
- Lavin M, Aguila S, Dalton N, Nolan M, Byrne M, Ryan K, et al. Significant gynecological bleeding in women with low von Willebrand factor levels. *Blood Adv.* 2018 Jul 24; 2(14):1784-1791.
- Halimeh S. Menorrhagia and postpartum haemorrhage in women with rare bleeding disorder. *Thromb Res.* 2015 Feb; 135 Suppl 1:S34-7.
- Gresele P, Falcinelli E, Bury L. Inherited platelet disorders in women. *Thromb Res.* 2019 Sep; 181 Suppl 1:S54-S59.
- DeLoughery E, Bannow BS. Anticoagulant therapy for women: implications for menstruation, pregnancy, and lactation. *Hematology Am Soc Hematol Educ Program.* 2022 Dec 9; 2022(1):467-473.

Screening Tools for Heavy Menstrual Bleeding

Recommended Reading

- ACOG Committee Opinion 651: Menstruation in girls and adolescents: using the menstrual cycle as a vital sign. *Obstet Gynecol*. 2015; 126:e143-6.
- ACOG Committee Opinion 785: Screening and Management of Bleeding Disorders in Adolescents with Heavy Menstrual Bleeding. *Obstet Gynecol*. 2019; 134:e71-e83.
- Woods K, Kearney S. Screening Tools for Evaluating the Bleeding Adolescent. In: Srivaths LV (eds) *Hematology in the Adolescent Female*. 1st ed. Springer, 2020:13-27.
- Zia A, Stanek J, Christian-Rancy M, Ahuja SP, Savelli S, O'Brien SH. Utility of a screening tool for haemostatic defects in a multicentre cohort of adolescents with heavy menstrual bleeding. *Haemophilia.* 2018 November; 24(6):957–963. doi:10.1111/hae.13609. Epub 2018 Sep 12. PMID: 30207633
- O'Brien SH. Evaluation and management of heavy menstrual bleeding in adolescents: the role of the hematologist. *Hematology Am Soc Hematol Educ Program.* 2018; (1):390–398. https://doi.org/10.1182/asheducation-2018.1.390

Supplemental Reading

- Borzutzky C, Jaffray J. Diagnosis and management of heavy menstrual bleeding and blood disorders in adolescents. *JAMA Pediatrics.* 2020; 174:186-94.
- Jain S, Zhang S, Acosta M, Malone K, Kouides P, Zia A. Prospective evaluation of ISTH-BAT as a predictor of bleeding disorder in adolescents presenting with heavy menstrual bleeding in a multidisciplinary hematology clinic. *J Thromb Haemost.* 2020 Oct; 18(10):2542-2550. doi: 10.1111/jth.14997. Epub 2020 Aug 28. PMID: 32654321
- Zia A, Kouides P, Khodyakov D, Dao E, Lavin M, Kadir RA, Othman M, Bauman D, Halimeh S, Winikoff R, Revel-Vilk S. Standardizing care to manage bleeding disorders in adolescents with heavy menses-A joint project from the ISTH pediatric/neonatal and women's health SSCs. *J Thromb Haemost.* 2020 Oct; 18(10):2759-2774. doi: 10.1111/jth.14974. PMID: 32573942
- Liberty A, Bannow B, Matteson K, Adelman A, Colwill A. Menstrual technology innovations and the implications for heavy menstrual bleeding. *Obstet Gynecol.* 2023 Apr; 141(4): 666-73.

Diagnostic Evaluation of Heavy Menstrual Bleeding in Adolescents

Recommended Reading

- Borzutzky C, Jaffray J. Diagnosis and management of heavy menstrual bleeding and blood disorders in adolescents. *JAMA Pediatrics.* 2020; 174:186-94.
- ACOG Committee Opinion 785: Screening and Management of Bleeding Disorders in Adolescents with Heavy Menstrual Bleeding. *Obstet Gynecol.* 2019; 134:e71-e83.
- O'Brien SH. Evaluation and management of heavy menstrual bleeding in adolescents: the role of the hematologist. *Blood.* 2018 Nov 15; 132(20):2134-2142. doi: 10.1182/blood-2018-05-848739. Epub 2018 Nov 14. PMID: 30429157.

Supplemental Reading

- Davila J, Alderman EM. Heavy menstrual bleeding in adolescent girls. *Pediatr Ann.* 2020 Apr 1; 49(4):e163-e169. doi: 10.3928/19382359-20200321-01. PMID: 32275760.

Diagnostic Evaluation of Heavy Menstrual Bleeding in Adults

Recommended Reading

- James AH, Kouides PA, Abdul-Kadir R, Dietrich JE, Edlund M, Federici AB et al. Evaluation and management of acute menorrhagia in women with and without underlying bleeding disorders: consensus from an international expert panel. *Eur J Obstet Gynecol Reprod Bio.l* 2011;158:124-34.
- ACOG Practice Bulletin 128: Diagnosis of Abnormal Uterine Bleeding in Reproductive-Aged Women. *Obstet Gynecol*. 2012; 120:197-206.

Supplemental Reading

- James AH. Heavy menstrual bleeding: work-up and management. *Hematology Am Soc Hematol Educ Program.* 2016; (1):236-42.

Laboratory Testing for Bleeding Disorders

Recommended Reading

- Borzutzky C, Jaffray J. Diagnosis and management of heavy menstrual bleeding and blood disorders in adolescents. *JAMA Pediatrics.* 2020; 174:186-94.
- James AH, Kouides PA, Abdul-Kadir R, Dietrich JE, Edlund M, Federici AB et al. Evaluation and management of acute menorrhagia in women with and without underlying bleeding disorders: consensus from an international expert panel. *Eur J Obstet Gynecol Reprod Biol.* 2011; 158:124-34.
- James PD, Connell NT, Ameer B, Di Paola J, Eidenboom J, Giraud N et al. ASH ISTH NHF WFH 2021 Guidelines on the diagnosis of von Willebrand disease. *Blood Adv.* 2021; 5:280-300.

Supplemental Reading

- Gomez K, Anderson J, Baker P, Biss T, Jennings I, Lowe G, Platton S, et al. Clinical and laboratory diagnosis of heritable platelet disorders in adult and children: a British Society for Haematology Guideline. *BJH.* 2021; 195:46-72.

- Thomas W, Downes K, Desborough MJR. Bleeding of unknown cause and unclassified bleeding disorders; diagnosis, pathophysiology and management. *Haemophilia.* 2020; 26:946-57.
- Dickerson KE, Menon N. Zia A. Abnormal Uterine Bleeding in Young Women with Blood Disorders. *Pediatr Clin North Am.* 2018; 65(3):543-60.

Additional Learning Materials

FWGBD Webinar: Rare Bleeding Disorder Manifestations in Women and Girls.

Screening for Iron Deficiency Anemia

Recommended Reading

- Cooke AG, McCavit TL, Buchanan GR, Powers JM. Iron deficiency anemia in adolescents who present with heavy menstrual bleeding. *J Pediatr Adolesc Gynecol.* 2017 Apr;30(2):247-250. doi: 10.1016/j.jpag.2016.10.010. Epub 2016 Oct 24. PMID: 27789349.
- Johnson S, Lang A, Sturm M, O'Brien SH. Iron Deficiency without anemia: A common yet under-recognized diagnosis in young women with heavy menstrual bleeding. *J Pediatr Adolesc Gynecol*. 2016 Dec; 29(6):628-631. doi: 10.1016/j.jpag.2016.05.009. Epub 2016 Jun 1. PMID: 27262832.

Oral and IV Iron Therapy for Iron Deficiency Anemia

Recommended Reading

- Ning S, Zeller MP. Management of Iron Deficiency. *Hematology Am Sco Hematol Educ Program.* 2019 Dec 6; 2019 (1):315-322. PMID: 31808874.
- Percy L, Mansour D, Fraser I. Iron deficiency and iron deficiency anemia in women. *Best Practice & Research: Clin Obstet & Gynaeco.l* 2017 Apr; 40:55-67.
- Mansour D, Hofman A, Gemzell-Danielsson K. A review of clinical guidelines on the management of iron deficiency and iron deficiency anemia in women with heavy menstrual bleeding. *Adv Ther.* 2021; 38(1):201-25.

Supplemental Reading

- Moretti D, Goede JS, Zeder C, et al. Oral iron supplements increase hepcidin and decrease iron absorption from daily or twice-daily doses in iron-depleted women. *Blood.* 2015; 126(17):1981-9.
- Powers JM, Shamoun M, McCavit TL, Adix L, Buchanan GR. Intravenous ferric carboxymaltose with iron deficiency anemia who respond poorly to oral iron. *J of Pediatrics.* 2017; 180:212-216. PMID: 27776750.

Additional Learning Materials

- FWGBD Case Conversation: Iron Deficiency and Fatigue Among Adolescents with Bleeding Disorders
- FWGBD Webinar: Iron Deficiency Anemia in Pregnancy
- FWGBD Webinar: Iron Deficiency Anemia and Heavy Menstrual Bleeding: Prevalence, Impact and Management

Hormonal Management of Heavy Menstrual Bleeding in Adolescents and Adults

Recommended Reading

- Baldwin MK, Bercaw-Pratt JL. Chapter 10: Hormonal Therapy for Heavy Menstrual Bleeding. In: Srivaths LV (eds) *Hematology in the Adolescent Female*. 1st ed. Springer, Cham; 2020: 109-118.
- Rodriguez MB, Lethaby A, Jordan V. Progestogen-releasing intrauterine systems for heavy menstrual bleeding. *Cochrane Database Syst Rev.* 2020; 6.
- Bryant-Smith AC, Lethaby A, Farquhar C, Hickey M. Antifibrinolytics for heavy menstrual bleeding. *Cochrane Database Syst Rev.* 2018; 4.

Supplemental Reading

- Boonyawat K, O'Brien SH, Bates SM. How I treat heavy menstrual bleeding associated with anticoagulants. *Blood.* 2017 Dec 14; 130(24):2603-2609.

Additional Learning Materials

- 7-2-1 Podcast: Demystifying IUDs (Part 1)
- 7-2-1 Podcast: Demystifying IUDs (Part 2)
- FWGBD Webinar: Addressing the use of Long-Acting Reversible Contraception (LARC), with Special Considerations for use with Young Women and Women with Bleeding Disorders

Non-Hormonal Management of Heavy Menstrual Bleeding in Adolescents and Adults

Recommended Reading

- Ahuja SP, Recht M, Konkle B. Chapter 11: Hemostatic Therapies for Heavy Menstrual Bleeding. In: Srivaths LV (eds) *Hematology in the Adolescent Female.*1st ed. Springer, Cham; 2020: 119-127.
- Bryant-Smith AC, Lethaby A, Farquhar C, Hickey M. Antifibrinolytics for heavy menstrual bleeding. *Cochrane Database Syst Rev.* 2018 Apr 15; 4(4):CD000249. doi: 10.1002/14651858.CD000249.pub2. PMID: 29656433; PMCID: PMC6494516.
- Rodeghiero F. Management of menorrhagia in women with inherited bleeding disorders: general principles and use of desmopressin. *Haemophilia.* 2008 Jan; 14 Suppl 1:21-30. doi: 10.1111/j.1365-2516.2007.01611.x. PMID: 18173691. https://onlinelibrary.wiley.com/doi/10.1111/j.1365-2516.2007.01611.x
- Ragni MV, Machin N, Malec LM, James AH, Kessler CM, Konkle BA, Kouides PA, Neff AT, Philipp CS, Brambilla DJ. Von Willebrand factor for menorrhagia: a survey and literature review. *Haemophilia.* 2016 May; 22(3):397-402. Doi: 10.1111/hae.12898. Epub 2016 Feb 4. PMID: 26843404; PMCID: PMC4874860.

Supplemental Reading

- Lumsden MA, Wedisinghe L. Tranexamic acid therapy for heavy menstrual bleeding. *Expert Opin Pharmacother*. 2011 Sep; 12(13):2089-95. Doi: 10.1517/14656566.2011.598857. Epub 2011 Jul 18. PMID: 21767224.
- Srivaths LV, Dietrich JE, Yee DL, Sangi-Haghpeykar H, Mahoney D. Oral tranexamic acid versus combined oral contraceptives for adolescent heavy menstrual bleeding: A pilot study. *J Pediatr Adolesc Gynecol.* 2015 Aug; 28(4):254-7. doi: 10.1016/j.jpag.2014.12.012. Epub 2014 Dec 29.PMID: 26024940

- Kadir RA, Lee CA, Sabin CA, Pollard D, Economides DL. DDAVP nasal spray for treatment of menorrhagia in women with inherited bleeding disorders: a randomized placebo-controlled crossover study. *Haemophilia.* 2002 Nov; 8(6):787-93.
- Naoulou B, Tsai MC. Efficacy of tranexamic acid in the treatment of idiopathic and non-functional heavy menstrual bleeding: a systematic review. *Acta Obstet Gynecol Scand.* 2012 May; 91(5):529-37. doi: 10.1111/j.1600-0412.2012.01361.x. Epub 2012 Feb 24. PMID: 22229782.
- Mauser-Bunschoten EP, Kadir RA, Laan ETM, Elfvinge P, Haverman L, Teela L, Degenaar MEL, Fransen van de Putte DE, D'Oiron R, van Galen KPM. Managing women-specific bleeding in inherited bleeding disorders: A multidisciplinary approach. *Haemophilia.* 2021 May; 27(3):463-469. doi: 10.1111/hae.14221. Epub 2020 Dec 13. PMID: 33314402
- Batsuli G, Kouides P. Rare Coagulation Factor Deficiencies (Factors VII, X, V, and II). *Hematol Oncol Clin North Am.* 2021 Dec; 35(6):1181-1196. doi: 10.1016/j.hoc.2021.07.010. Epub 2021 Aug 10. PMID: 34389198.

Additional Learning Materials

- FWGBD Webinar: Achieving Hemostasis in Women with Bleeding Disorders: Challenges and Therapies in VWD
- FWGBD Webinar: Morbidity Challenges and Treatment Advances for Hemophilia Carriers

Heavy Menstrual Bleeding and Quality of Life

Recommended Reading

- Pawar A, Rulkarni R. Chapter 12: Quality of Life in Female Adolescents with Bleeding Disorders. In: Srivaths LV (eds). *Hematology in the Adolescent Female*. 1st ed. Springer, Cham; 2020:129-137.
- Klein DA, Goldenring JM, Adelman WP. HEEADSSS 3.0 The psychosocial interview for adolescents updated for a new century fueled by media. *Contemporary Pediatrics*. January 2014:1-16.

Supplemental Reading

- Marcell AV, Burstein GR. Sexual and Reproductive Health Care Services in the Pediatric Setting. *American Academy of Pediatrics.* 2017; 140:1-13.
